# Tomatoes for Supper

**Published:** 1864-10

**Authors:** 


### TOMATOES FOR SUPPER.

The scissoring editor who printed the following ought to be kicked and the lady writer ought to be kickter.

" For a family of half a dozen persons, take six eggs, boil four of them *hard*, dissolve the yolks with vinegar sufficient, add about three teaspoons of mustard and mash as smooth as possible ; then add the two remaining eggs, (raw,) yolk and white, stir well ; then add salad oil to make altogether sauce sufficient to cover the tomatoes well ; add plenty of salt and cayenne pepper, and beat thoroughly until it frosts. Skin and cut the tomatoes a full fourth of an inch thick, and pour the sauce over, and you have a dish fit for a President."

That is the whole story, and what a mess of eggs and mustard and salt and cayenne pepper, it is enough to sicken a horse or choak an elephant ; and then to call it a dish of tomatoes when not the slightest intimation is given as to the amount of tomatoes to be used, whether a grain or a teaspoonful. And to think that any woman could relish such a lob-lolly ; why the veriest drunkards are the persons who revel in red pepper, vinegar and brimstone after a week's spree. And this recipe first saw the light of day, in print, when eggs were selling in New York at four cents a piece, and tomatoes at twenty-five cents a mouthful, for if you were to squeeze all the water out of a quart of tomatoes there would not be a tablespoonful left. If great big strapping country girls who can stomach such a bowl of slosh want to make themselves useful, they will serve their day and generation a thousand times better by studying out some cheap method of making corn bread taste as good as pound cake ; of preparing as luscious a meal out of liver as from porter house steak. If any maid, miss or mistress can invent a plan for making as good a cup of coffee out of burnt bread crusts as from the best old government java, or can make "catnip tea" without sugar, taste as well and exhilarate more than the best "store" article, such are the persons who merit the thanks and respect of the nation. How could the editor of the Germantown Telegraph, which has been so well conducted for now these many years, have allowed such a jumble to mar his fair columns. The letter which brought it from his fair correspondent must have been gilt edged, rose-scented and handed in by a waiter in livery, just at the moment of his learning from another missive that a New Master Telegraph had just safely walked out upon the boards of this mundane sphere, and perhaps too, he had just heard from his paper maker that "stock" had fallen twenty-eight-and-a-half per cent. Some such streak of good luck must have come over him to have put him in the mood of sending up for copy that squashy article headed "Tomatoes for Supper." The fact is no body ought to have any supper except old women and babies. Whats the use of eating from morning till night. Breakfast, Lunch, Dinner ; Supper—Tea ; no wonder a hundred people died every day last week in New York City, every one of them ought to have had a "coroner's inquest." No doubt the just verdict would have been as to ninety-nine out of a hundred "died of a surfeit of food—or physic !" No, let the newspapers hold out inducements to correspondents to communicate information how the cheapest articles of food can be made to taste the most luscious, and thus merit a wider patronage.

